# A game-theoretic model of Monkeypox to assess vaccination strategies

**DOI:** 10.7717/peerj.9272

**Published:** 2020-06-22

**Authors:** Sri Vibhaav Bankuru, Samuel Kossol, William Hou, Parsa Mahmoudi, Jan Rychtář, Dewey Taylor

**Affiliations:** 1 Department of Biomedical Engineering, Virginia Commonwealth University, Richmond, VA, United States of America; 2 Department of Kinesiology and Health Sciences, Virginia Commonwealth University, Richmond, VA, United States of America; 3 Department of Biology, Virginia Commonwealth University, Richmond, VA, United States of America; 4 Department of Mathematics and Applied Mathematics, Virginia Commonwealth University, Richmond, VA, United States of America

**Keywords:** Monkeypox, Game theory, Nash equilibrium, Smallpox, Vaccination

## Abstract

Monkeypox (MPX) is a zoonotic disease similar to smallpox. Its fatality rate is about 11% and it is endemic to the Central and West African countries. In this paper, we analyze a compartmental model of MPX dynamics. Our goal is to see whether MPX can be controlled and eradicated by voluntary vaccinations. We show that there are three equilibria—disease free, fully endemic and previously neglected semi-endemic (with disease existing only among humans). The existence of semi-endemic equilibrium has severe implications should the MPX virus mutate to increased viral fitness in humans. We find that MPX is controllable and can be eradicated in a semi-endemic equilibrium by vaccination. However, in a fully endemic equilibrium, MPX cannot be eradicated by vaccination alone.

## Introduction

Monkeypox (MPX) is a zoonotic disease that has the potential to develop into one of the most threatening human *Orthopoxvirus* infections since the eradication of smallpox (Durski et al., 2018). The causative agent of MPX is monkeypox virus (MPXV), found in the same genus as the variola virus (smallpox), vaccinia virus, and cowpox virus (Shchelkunov, Marennikova & Moyer, 2006; Sklenovská & Van Ranst, 2018). Common symptoms of MPX, though relatively milder than smallpox, include fever, severe headaches, skin lesions, and myalgia (CDC, 2003). Prevention of the disease has remained a challenge for poverty-stricken rural areas with poor infrastructure that lack necessary sanitary supervision (Sklenovská & Van Ranst, 2018).

MPX is endemic to Central Africa and West Africa (Weinstein et al., 2005; Yinka-Ogunleye et al., 2018). West African and Central African strains of MPXV exist, the latter of which is more virulent and symptomatically severe (Likos et al., 2005; Mwamba et al., 2014). In the Democratic Republic of the Congo (DRC) the mortality rate of the Central African strain is 11% (Ježek et al., 1987). Since the first case of human infection in 1970, there have been numerous outbreaks in the DRC (Eteng et al., 2018). Annually, the DRC reports over 2,000 cases of suspected infections and is the only country in an endemic state (Mwamba et al., 2014). This estimate may be modest, as MPX is often misdiagnosed as chickenpox or other diseases that cause rashes (Ježek et al., 1988). Additionally, modern and robust surveillance of MPX is neglected as a consequence of limited funds and resources (Rimoin et al., 2010), and countries other than the DRC are not required to report all cases of MPX (Durski et al., 2018). Thus, the disease may be more severe than previously estimated.

In 2003, 47 cases of MPX were reported across five states in the U.S., originating from a shipment of animals from Ghana imported to Texas (CDC, 2003). In 2018, 3 cases of MPX were reported in the United Kingdom, making it the first time since the 2003 United States outbreak that the disease had reached a country outside of Africa (Eteng et al., 2018).

The clinical presentation of MPX can be found in Di Giulio & Eckburg (2004). The incubation period for the virus ranges from five to 21 days. MPX infection is split into two distinct phases: the invasion period and the skin eruption period. The invasion period starts between 0–5 days and is characterized by fever, lymphadenopathy, intense asthenia, severe headaches, and myalgia. The skin eruption period occurs 1–3 days after the appearance of a fever or lymphadenopathy, and it is characterized by rash formation, which often begins on the face and spreads to the rest of the body. The rash first appears as maculopapules (lesions with flat bases) and progresses to fluid filled blisters called vesicles. The vesicles then burst, forming pustules, and a crust forms over the affected area within 10 days. The number of lesions formed can vary from a few to thousands across the body, with children reportedly experiencing more severe symptoms than adults.

The predominant mode of MPX transmission is through human-animal interaction. Direct contact with an infected animal’s blood, bodily fluids, or lesions can lead to infection. Documented cases of MPX in Central and West Africa show that transmission can occur via the handling of wild animals, predominantly monkeys (Reynolds et al., 2017). Cultural influences, such as consumption of “bush meat,” can be a potential source of transmission. Additionally, direct contact with an infected person’s bodily fluids and skin lesions can lead to the transmission of the disease (McCollum & Damon, 2013).

Despite MPX’s high case fatality rate (Ježek et al., 1987) there are no known cures (Eteng et al., 2018). Until recently, there were no disease-specific preventative measures such as vaccines, though existing smallpox vaccines have historically been around 85% successful (Eteng et al., 2018). However, administration of the smallpox vaccine has ceased since the disease’s eradication in 1980, resulting in lowered immunity against *Orthopoxviruses* in general. This has led to a supposed increase in population susceptibility to MPXV (Sklenovská & Van Ranst, 2018). In 2019, the vaccine termed Jynneos^®^ was approved by the US FDA for protection against VARV and MPXV (Meyer, Ehmann & Smith, 2020).

It is possible that this lack of preventative measures is partially explained by a matching lack of literature on the potential dangers of inter-human transmission of MPX (Rimoin et al., 2010; Mwamba et al., 2014; Doshi et al., 2018; Sklenovská & Van Ranst, 2018). The urgency for better research on MPX is exacerbated considering vaccination cessation and immunocompromised populations in Central Africa, so a need for comprehensive preventative strategies is apparent (WHO, 2017). The factors such as (1) a lack of an effective vaccination strategy from fixed bases, (2) a shortfall in the vaccine supply and (3) logistical and security problems associated with the distance from the health centers, all contribute to the challenges of vaccinating the whole population in Central and Western Africa (Herp et al., 2003).

The identity of MPXV reservoir host(s) remains unknown (Di Giulio & Eckburg, 2004; Falendysz et al., 2017). The seroprevalence of MPXV was found highest in a population of moribund rope squirrels (*Funisciurus anerythrus*) in Zaire (now DRC), and the virus was also found in sun squirrels (*Heliosciurus rufobrachium*) and non-human primates in DRC (Khodakevich et al., 1987; Khodakevich, Ježek & Messinger, 1988) as well as in Gambian pouched rats (*Cricetomys gambianus*) (Doty et al., 2017; Doshi et al., 2019). In West Africa, African dormice (*Graphiurus sp.*) and ground squirrels (*Xerus sp.*) were identified as additional hosts (Reynolds et al., 2010). The majority of reported human cases originate from an interaction with an infected animal (Arita et al., 1985). The transmission of MPX among animals can be affected by environmental conditions (Brown & Leggat, 2016). Deforestation and flooding could potentially increase or decrease the MPX reservoirs, depending on how the animal population is affected by these conditions (Brown & Leggat, 2016). Long-distance transportation of potential MPX carriers may result in the expansion of the geographical range of the MPX reservoir, as exemplified by the 2003 US outbreak.

Currently, MPXV likely needs the animal reservoir as the human-to-human transmission chains of MPX are relatively short; the maximum number of generations reported in literature is seven (Learned et al., 2005). Nevertheless, as demonstrated by the case of H1N1 influenza (swine flu), some virus mutations can increase viral fitness in humans (Elderfield et al., 2014). We note that poxviruses have linear, double-stranded DNA genomes that vary from 130 to 230 kbp (Moss, 2013) and as such are evolving much slower than H1N1. Nevertheless, they can still adapt rapidly (Elde et al., 2012) and genetic engineering and modern molecular biology already turned a mousepox virus into an unusually lethal strain (Jackson et al., 2001; Di Giulio & Eckburg, 2004).

Epidemiologic compartmental models have been used to better understand the potential implications of disease transmission and infection (Blackwood & Childs, 2018; Bidari & Goldwyn, 2019). For MPX, the framework for a mathematical model has been tentatively set, but existing iterations have had shortcomings, failing to address some of the aforementioned aspects of the disease in their entirety. Bhunu & Mushayabasa (2011) introduced a basic SIR vector-borne compartmental model between humans and primates, yet deem an endemic state solely in humans as trivial. Usman & Adamu (2017) build upon this framework by introducing an SVEIR compartmental model to account for the disease’s incubation period and potential vaccine.

Game theoretical models attempt to study complex scenarios in which self-interested individuals will take an action based on the decisions of the rest of the population (Bauch & Earn, 2004). The model is a predictive tool in populations for extracting an optimum decision-making strategy (Chang et al., 2020). Game theory has been applied to protection strategies to control diseases such as smallpox (Bauch, Galvani & Earn, 2003), toxoplasmosis (Sykes & Rychtář, 2015), cholera (Kobe et al., 2018), measles (Shim et al., 2012), rubella (Shim, Kochin & Galvani, 2009), influenza (Galvani, Reluga & Chapman, 2007), African sleeping sickness (Crawford et al., 2015), malaria (Orwa, Mbogo & Luboobi, 2018; Broom, Rychtář & Spears-Gill, 2016), (Zika Padmanabhan, Seshaiyer & Castillo-Chavez, 2017; Banuelos et al., 2019) (Polio Cheng et al., 2020), Ebola (Brettin et al., 2018), chikungunya (SRM Klein, AO Foster, DA Feagins, JT Rowell, IV Erovenko, 2019, unpublished data), meningitis (A Martinez, J Machado, E Sanchez, I Erovenko, 2019, unpublished data), typhoid (C Acosta-Alonzo, IV Erovenko, A Lancaster, H Oh, J Rychtář, D Taylor, 2020, unpublished data), Hepatitis C (Scheckelhoff, Ejaz & Erovenko, 2019) and Hepatitis B (Chouhan et al., in press) among others. In this paper, we apply a similar approach to MPX to investigate a scenario in which individuals have the option of vaccinating to reduce the chance of contracting the virus. We further evaluate vaccination strategies on an individual and population-wide level by discussing the vaccination rates required to achieve herd immunity and Nash equilibrium.

In the present study, we build on the work of Usman & Adamu (2017), see also Lauko, Pinter & TeWinkel (2018) for a simplified SIR version of the model. The mathematical model of the MPX dynamics is shown in the next section. Then, we provide closed-form formulas for equilibrium states of MPX dynamics; the formulas provided in Usman & Adamu (2017) do not allow for direct calculations of the equilibria. We also show the existence of a “semi-endemic” equilibrium. This was not previously discussed in Usman & Adamu (2017), although it appears in Lauko, Pinter & TeWinkel (2018). We apply a game-theoretic approach to evaluate individual and population-wide vaccination strategies on the basis of cost and probabilistic disease acquisition and then we perform sensitivity analysis. We conclude the study by a discussion.

## Mathematical Model

**Figure 1:**
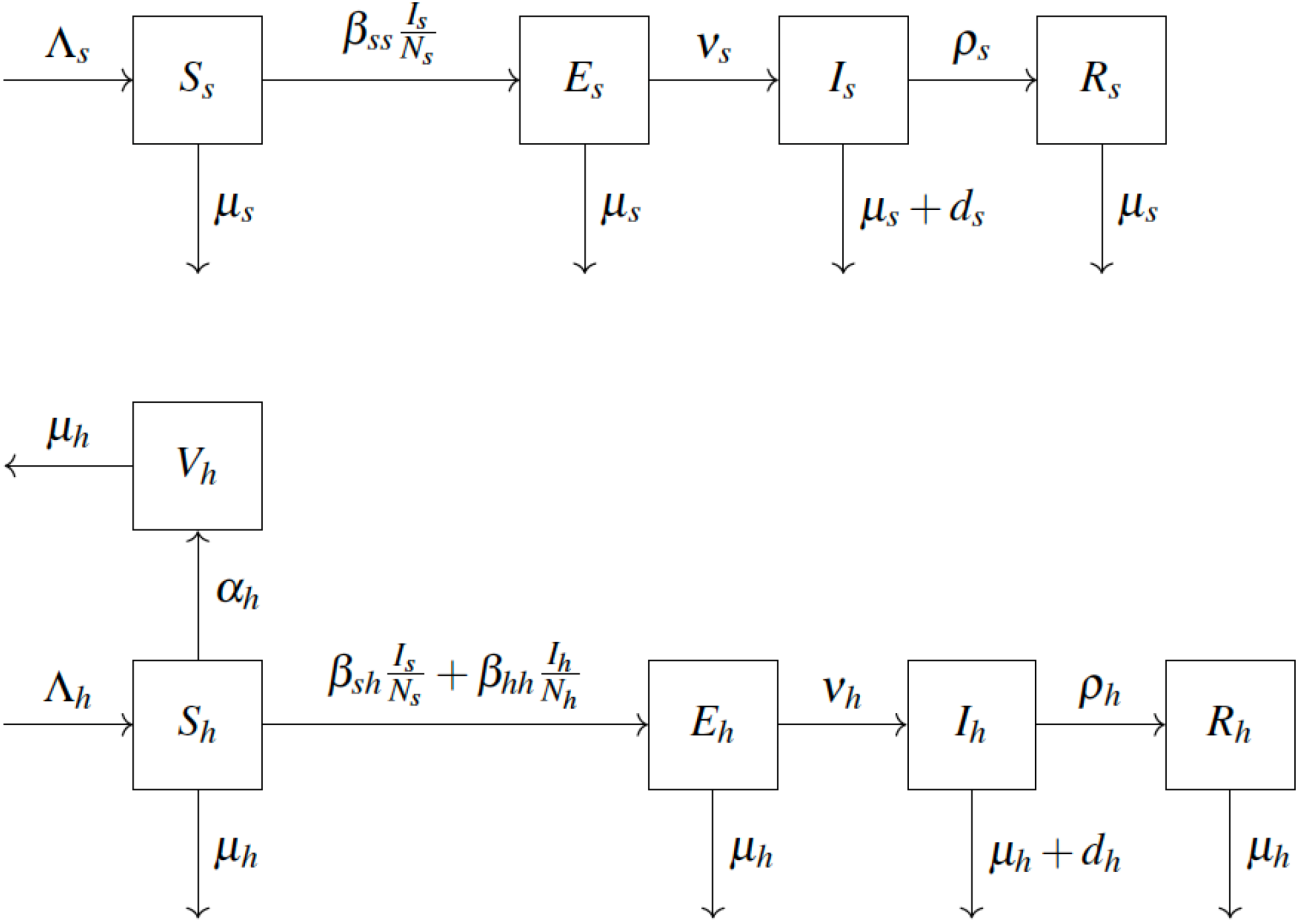
Scheme of mathematical model for humans and squirrels, adapted from Usman & Adamu (2017).

We adopt the compartmental epidemiological model introduced in Usman & Adamu (2017) and shown in Fig. 1. We consider squirrels to be the primary reservoir hosts. The population is divided into squirrels and humans, denoted by *s* and *h* subscripts, respectively. Individuals are born as Susceptible (*S*) at rate Λ. Susceptible humans vaccinate (move to *V*_*h*_) at rate *α*_*h*_. Vaccinated humans are assumed to never contract the disease in the remainder of their lifetime. Susceptible squirrels become Exposed (*E*_*s*_) by coming into contact with infected squirrels with effective transmission rate *β*_*ss*_. Susceptible humans become exposed by coming into contact with either infected squirrels (with effective transmission rate *β*_*sh*_) or infected humans (with effective transmission rate *β*_*hh*_). After an incubation period *ν*^−1^, the exposed individuals become Infected (*I*). Infected individuals are infectious. They Recover (*R*) at rate *ρ*. Any individual may die due to natural causes at rate µ. Infected individuals can also die from the disease at rate *d*. The notation is summarized in Table 1. The model yields the following differential equations, see for example Blackwood & Childs (2018).

**Table 1:** Model parameters. The human MPX related death rate was taken as a solution to *d*_*h*_∕(*d*_*h*_ + *ρ*_*h*_) = 0.1 where 10% is the MPX fatality (Ježek et al., 1987). Similarly, squirrel MPX related death rate was taken as a solution to *d*_*s*_∕(*d*_*s*_ + *ρ*_*s*_) = 0.6 where 60% is an estimate for the MPX fatality found between 50–75% (Falendysz et al., 2017). We estimated the effective squirrel-to-squirrel transmission rate as *β*_*ss*_ = 40; this yields about 24% of seropositive squirrels in the population, a number that agrees with estimates from Khodakevich, Ježek & Messinger (1988). The effective transmission rates between humans was estimated as *β*_*hh*_ = 32.85 as follows. Arita et al. (1985) provide transmission risk *p* = 0.15 amongst household contacts and *p* = 0.03 amongst other contacts. We assumed human-to-human contact rate *γ* = 365 (i.e., once a day) and obtained ${\beta }_{hh}=365 \frac{0.15+0.03}{2} =32.85$. The effective squirrel-to-human transmission rate was estimated to be *β*_*sh*_ = 0.05 as this yields about 1% of seropositive humans (Khodakevich, Ježek & Messinger, 1988). The actual cost of vaccine is $4.85 (Lambert de Rouvroit & Heegaard, 2016). While the vaccine is provided for free, there are many other direct and indirect costs associated with vaccination (need to travel to the health center, associated security risk, loss of income etc., see for example Herp et al. (2003)) and we estimated the cost of vaccination to be 4. We note that the previously approved smallpox vaccines such as ACAM2000 could cause severe side effects (Wollenberg & Engler, 2004; Nalca & Zumbrun, 2010). It is not clear if the new vaccine, JYNNEOS, is more effective to protect against MPXV infections in humans than ACAM2000 and what the potential side effects are.

Symbol	Meaning	Value	Source
Λ_*h*_	Human birth rate	0.0328	CIA (2019)
Λ_*s*_	Squirrel birth rate	2	Hayssen (2008)
*μ*_*h*_	Human natural death rate	1∕60	World Bank (2019)
*μ*_*s*_	Squirrel natural death rate	0.5	Khodakevich, Ježek & Messinger (1988)
*d*_*h*_	Human MPX related death rate	3.12	Ježek et al. (1987)
*d*_*s*_	Squirrel MPX related death rate	17.5	Falendysz et al. (2017)
*ρ*_*h*_	Human recovery rate	28.08	Di Giulio & Eckburg (2004)
*ρ*_*s*_	Squirrel recovery rate	12	Falendysz et al. (2017)
*ν*_*h*_	Human infection rate	30.42	Di Giulio & Eckburg (2004)
*ν*_*s*_	Squirrel infection rate	120	Falendysz et al. (2017)
*α*_*h*_	Vaccination rate	variable	
*β*_*ss*_	Squirrel-to-squirrel transmission rate	40	Assumed based on Khodakevich, Ježek & Messinger (1988)
*β*_*sh*_	Squirrel-to-human transmission rate	0.05	Assumed based on Khodakevich, Ježek & Messinger (1988)
*β*_*hh*_	Human-to-human transmission rate	32.85	Arita et al. (1985)
*C*_*V*_	Cost of vaccination	4	Herp et al. (2003)
*C*_*MPX*_	Cost of MPX infection	$100	Adam, Evans & Murray (2003)

(1) \begin{eqnarray*} \frac{d{S}_{s}}{dt} & ={\Lambda }_{s}- \left( {\mu }_{s}+{\beta }_{ss} \frac{{I}_{s}}{{N}_{s}} \right) {S}_{s}\end{eqnarray*}

(2) \begin{eqnarray*} \frac{d{E}_{s}}{dt} & ={\beta }_{ss} \frac{{I}_{s}}{{N}_{s}} {S}_{s}- \left( {\mu }_{s}+{\nu }_{s} \right) {E}_{s}\end{eqnarray*}

(3) \begin{eqnarray*} \frac{d{I}_{s}}{dt} & ={\nu }_{s}{E}_{s}-({\mu }_{s}+{d}_{s}+{\rho }_{s}){I}_{s}\end{eqnarray*}

(4) \begin{eqnarray*} \frac{d{R}_{s}}{dt} & ={\rho }_{s}{I}_{s}-{\mu }_{s}{R}_{s}\end{eqnarray*}

(5) \begin{eqnarray*} \frac{d{S}_{h}}{dt} & ={\Lambda }_{h}- \left( {\mu }_{h}+ \left( {\beta }_{sh} \frac{{I}_{s}}{{N}_{s}} +{\beta }_{hh} \frac{{I}_{h}}{{N}_{h}} \right) +{\alpha }_{h} \right) {S}_{h}\end{eqnarray*}

(6) \begin{eqnarray*} \frac{d{V}_{h}}{dt} & ={\alpha }_{h}{S}_{h}-{\mu }_{h}{V}_{h}\end{eqnarray*}

(7) \begin{eqnarray*} \frac{d{E}_{h}}{dt} & = \left( {\beta }_{sh} \frac{{I}_{s}}{{N}_{s}} +{\beta }_{hh} \frac{{I}_{h}}{{N}_{h}} \right) {S}_{h}-({\mu }_{h}+{\nu }_{h}){E}_{h}\end{eqnarray*}

(8) \begin{eqnarray*} \frac{d{I}_{h}}{dt} & ={\nu }_{h}{E}_{h}-({\mu }_{h}+{d}_{h}+{\rho }_{h}){I}_{h}\end{eqnarray*}

(9) \begin{eqnarray*} \frac{d{R}_{h}}{dt} & ={\rho }_{h}{I}_{h}-{\mu }_{h}{R}_{h}\end{eqnarray*}

## Results

### Equilibrium states of the MPX dynamics

The basic reproduction numbers were derived by Usman & Adamu (2017) and are given by

(10) \begin{eqnarray*}{R}_{0ss}& ={\beta }_{ss}\cdot \frac{1}{{\mu }_{s}+{d}_{s}+{\rho }_{s}} \cdot \frac{{\nu }_{s}}{{\mu }_{s}+{\nu }_{s}} \end{eqnarray*}

(11) \begin{eqnarray*}{R}_{0hh}& ={\beta }_{hh}\cdot \frac{{\mu }_{h}}{{\alpha }_{h}+{\mu }_{h}} \cdot \frac{1}{{\mu }_{h}+{d}_{h}+{\rho }_{h}} \cdot \frac{{\nu }_{h}}{{\mu }_{h}+{\nu }_{h}} .\end{eqnarray*}

As shown in Appendix 1, *R*_0*ss*_ corresponds to a number of secondary squirrel infections caused by a single infected squirrel in an otherwise healthy population. The meaning of *R*_0*hh*_ is similar.

There are three qualitatively distinct equilibria of the dynamics (1)–(9). First, *ϵ*^0^ is the disease free equilibrium. It occurs when *R*_0*ss*_ < 1 and *R*_0*hh*_ < 1. Second, *ϵ*^∗^ is the fully endemic equilibrium with disease occurring amongst humans as well as squirrels. The equilibrium is stable when *R*_0*ss*_ > 1. Finally, *ϵ*^†^ is a semi-endemic equilibrium with disease prevalent only amongst human population. It is stable when *R*_0*ss*_ < 1 and *R*_0*hh*_ > 1.

The closed form formulas are given in Table 2. Step-by-step derivation can be found in Appendix 1.

**Table 2:** Different equilibria of the MPX dynamics. The formulas for ${N}_{h}^{\ast }$ and ${N}_{h}^{\dagger }$ are too long for the table and are given in Appendix 1.

	Disease-free (*ϵ*^0^)	Fully endemic (*ϵ*^∗^)	Semi-endemic (*ϵ*^†^)
*N*_*s*_	$ \frac{{\Lambda }_{s}}{{\mu }_{s}} $	$ \frac{{\Lambda }_{s}. \left( \frac{{\mu }_{s}+{d}_{s}+{\rho }_{s}}{{\nu }_{s}} +1+ \frac{{\rho }_{s}}{{\mu }_{s}} \right) }{{\mu }_{s}. \left( \frac{{\mu }_{s}+{d}_{s}+{\rho }_{s}}{{\nu }_{s}} \right) +{d}_{s}. \left( 1- \frac{1}{{R}_{0ss}} \right) +{\mu }_{s}+{\rho }_{s}} $	$ \frac{{\Lambda }_{s}}{{\mu }_{s}} $
*S*_*s*_	${N}_{s}^{0}$	${N}_{s}^{\ast }. \frac{1}{{R}_{0ss}} $	${N}_{s}^{\dagger }$
*E*_*s*_	0	$ \left( \frac{{\mu }_{s}+{d}_{s}+{\rho }_{s}}{{\nu }_{s}} \right) .{I}_{s}^{\ast }$	0
*I*_*s*_	0	$ \frac{{\Lambda }_{s}-{\mu }_{s}{N}_{s}^{\ast }}{{d}_{s}} $	0
*R*_*s*_	0	$ \frac{{\rho }_{s}}{{\mu }_{s}} .{I}_{s}^{\ast }$	0
*N*_*h*_	$ \frac{{\Lambda }_{h}}{{\mu }_{h}} $	(45)	(59)
*S*_*h*_	$ \frac{{\mu }_{h}}{{\mu }_{h}+{\alpha }_{h}} .{N}_{h}^{0}$	$ \frac{({\mu }_{h}+{\nu }_{h}) \left( \frac{{\mu }_{h}+{d}_{h}+{\rho }_{h}}{{\nu }_{h}} \right) .{I}_{h}^{\ast }}{{\beta }_{sh} \left( \frac{{I}_{s}^{\ast }}{{N}_{s}^{\ast }} \right) +{\beta }_{hh} \left( \frac{{I}_{h}^{\ast }}{{N}_{h}^{\ast }} \right) } $	$ \frac{ \left( {\mu }_{h}+{d}_{h}+{\rho }_{h} \right) \left( {\mu }_{h}+{\nu }_{h} \right) }{{\nu }_{h}{\beta }_{hh}} .{N}_{h}^{\dagger }$
*V*_*h*_	$ \frac{{\alpha }_{h}}{{\mu }_{h}+{\alpha }_{h}} .{N}_{h}^{0}$	$ \frac{{\alpha }_{h}}{{\mu }_{h}} .{S}_{h}^{\ast }$	$ \left( \frac{{\alpha }_{h}}{{\mu }_{h}} \right) .{S}_{h}^{\dagger }$
*E*_*h*_	0	$ \left( \frac{{\mu }_{h}+{d}_{h}+{\rho }_{h}}{{\nu }_{h}} \right) .{I}_{h}^{\ast }$	$ \left( \frac{{\mu }_{h}+{d}_{h}+{\rho }_{h}}{{\nu }_{h}} \right) .{I}_{h}^{\dagger }$
*I*_*h*_	0	$ \frac{{\Lambda }_{h}-{\mu }_{h}{N}_{h}^{\ast }}{{d}_{h}} $	$ \frac{{\Lambda }_{h}-{\mu }_{h}{N}_{h}^{\dagger }}{{d}_{h}} $
*R*_*h*_	0	$ \frac{{\rho }_{h}}{{\mu }_{h}} .{I}_{h}^{\ast }$	$ \frac{{\rho }_{h}}{{\mu }_{h}} .{I}_{h}^{\dagger }$

### Herd immunity and Nash equilibrium vaccination rates

The average cost of not vaccinating when the population vaccination rate is *α*_*h*_ is denoted *C*_*notV*_(*α*_*h*_) and it is given as a product of the cost of the MPX infection (*C*_*MPX*_) and the probability of moving from the *S*_*h*_ compartment to the *I*_*h*_ compartment, i.e.,

(12) \begin{eqnarray*}{C}_{notV}({\alpha }_{h})={C}_{MPX}\cdot \left( \frac{ \left( {\beta }_{sh} \frac{{I}_{s}}{{N}_{s}} +{\beta }_{hh} \frac{{I}_{h}}{{N}_{h}} \right) }{ \left( {\beta }_{sh} \frac{{I}_{s}}{{N}_{s}} +{\beta }_{hh} \frac{{I}_{h}}{{N}_{h}} \right) +{\mu }_{h}} \right) \cdot \left( \frac{{\nu }_{h}}{{\nu }_{h}+{\mu }_{h}} \right) .\end{eqnarray*}

As the vaccination rate *α*_*h*_ increases, *R*_0*hh*_ decreases by Eq. (11). Furthermore, the fraction $ \frac{{I}_{h}}{{N}_{h}} $ at the appropriate equilibrium also decreases. Consequently, the cost of not vaccinating decreases. In the semi-endemic equilibrium, the cost eventually becomes 0 when the vaccination rate reaches

(13) \begin{eqnarray*}{\alpha }_{HI}=\max \nolimits \left\{ 0, \frac{{\nu }_{h}{\beta }_{hh}{\mu }_{h}}{({\mu }_{h}+{d}_{h}+{\rho }_{h})({\mu }_{h}+{\nu }_{h})} -{\mu }_{h} \right\} .\end{eqnarray*}

**Figure 2:**
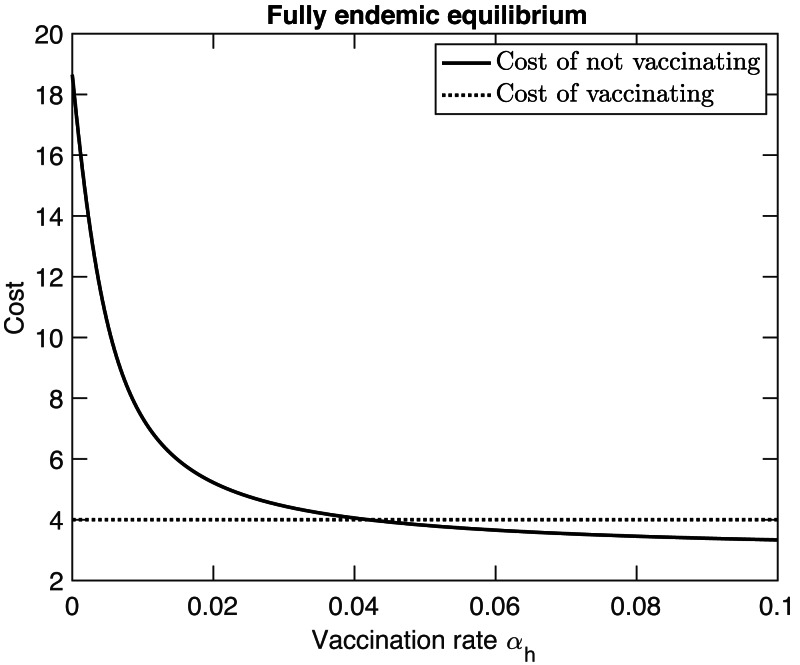
Cost versus vaccination rate. The vaccination rate (*α*_*h*_) is varied while all other parameter values are as specified in Table 1.

At that point, herd immunity is achieved and the disease is eradicated from the population. In the fully endemic equilibrium, there is always a reservoir of MPX in the squirrel population. This reservoir causes an influx of MPX infections amongst humans. Therefore the disease can never be fully eradicated and the cost of not vaccinating will never reach 0, see Fig. 2.

When the vaccination rate is such that *C*_*notV*_(*α*_*h*_) = *C*_*V*_, the vaccination rate is at Nash equilibrium, *α*_*NE*_. When *α*_*h*_ < *α*_*NE*_, it is beneficial for the individual to vaccinate; when *α*_*h*_ > *α*_*NE*_, it is beneficial for the individual not to vaccinate.

**Figure 3:**
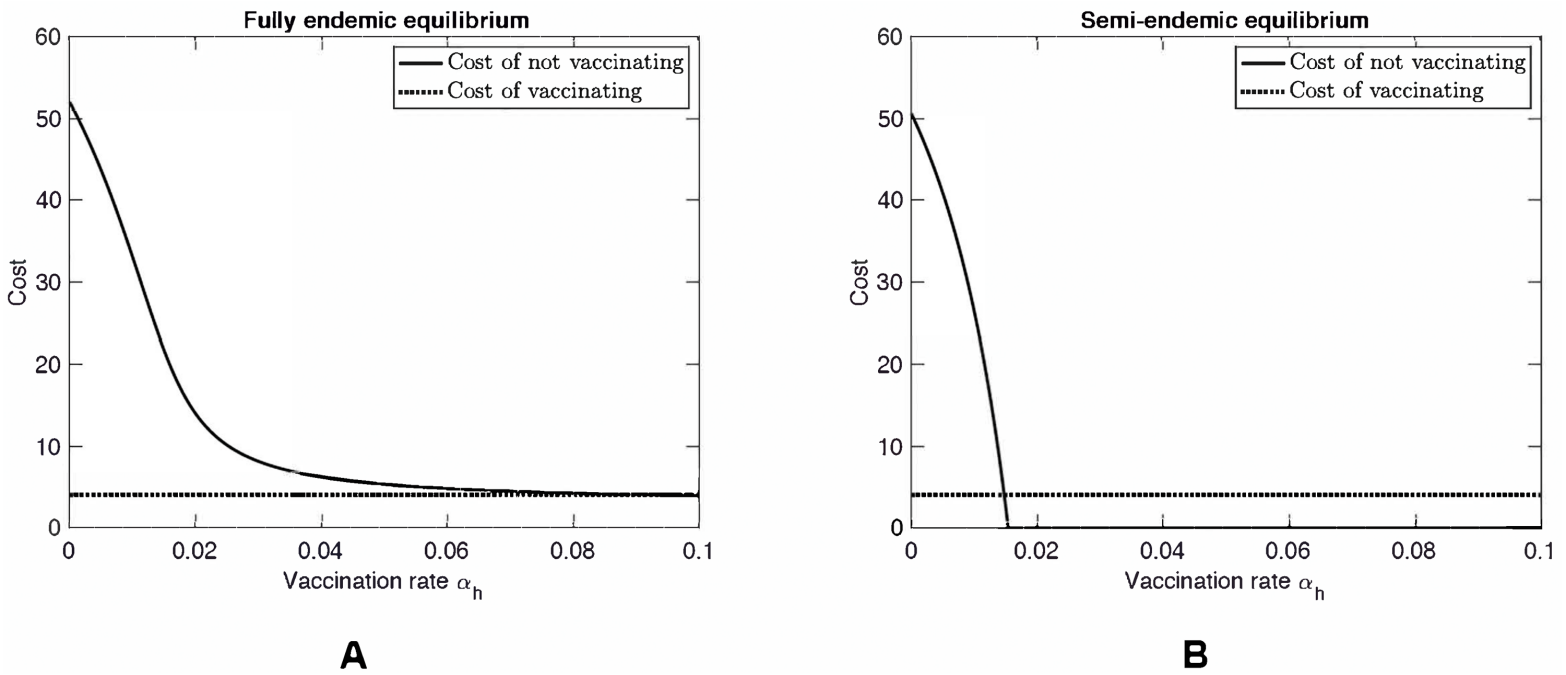
Costs vs. Vaccination rate when the effective human-to-human transmission rate is high, *β*_*hh*_ = 60. (A) The fully endemic state. (B) The semi-endemic state (*β*_*ss*_ = 30 $\lt ({\mu }_{s}+{d}_{s}+{\rho }_{s})\cdot \frac{{\mu }_{s}+{\nu }_{s}}{{\nu }_{s}} $). The same scenario occurs when *β*_*sh*_ = 0 and *β*_*ss*_ is arbitrary. In both figures, the vaccination rate (*α*_*h*_) is varied while all other parameter values are as specified in Table 1.

Figure 3 shows a scenario where *β*_*hh*_ = 60. While this value is unrealistically high, we investigated this hypothetical scenario to see what would happen if MPXV mutates as was the case of H1N1 influenza (swine flu) or is genetically engineered as was the case of mousepox (Jackson et al., 2001; Di Giulio & Eckburg, 2004). When *β*_*hh*_ is large enough, specifically when

(14) \begin{eqnarray*}{\beta }_{hh}\gt \frac{{\alpha }_{h}+{\mu }_{h}}{{\alpha }_{h}} \cdot ({\mu }_{h}+{d}_{h}+{\rho }_{h})\cdot \frac{{\mu }_{h}+{\nu }_{h}}{{\nu }_{h}} \end{eqnarray*}

MPX no longer needs squirrels to persist in the human population. In particular, it can become endemic even in countries without natural squirrel population (i.e., even when *β*_*sh*_ = 0). At the same time, in the semi-endemic equilibrium, the disease can be controlled through vaccination. Note that there is almost no difference between the Nash equilibrium rate *α*_*NE*_ (a solution to *C*_*notV*_(*α*_*h*_) = *C*_*V*_) and the rate *α*_*HI*_ needed for herd immunity (a solution to *C*_*notV*_(*α*_*h*_) = 0).

### Sensitivity analysis

**Figure 4:**
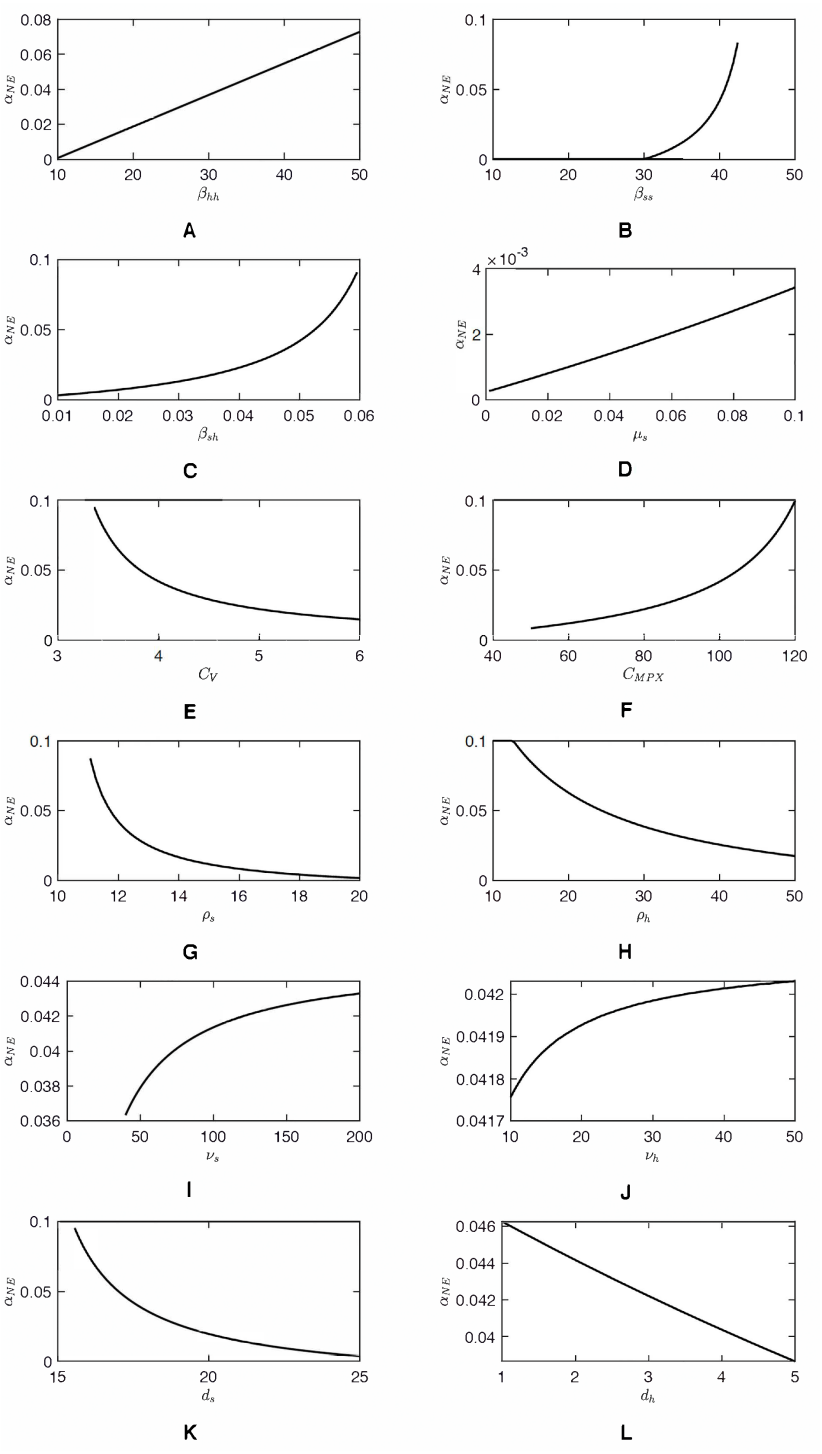
Dependence of *α*_*NE*_ on different parameter values (A–L). Unless varied, the parameter values are as specified in Table 1. For those parameters, *α*_*NE*_ = 0.0419 and the sensitivity index, $S{I}_{x}= \left( \frac{x}{{\alpha }_{NE}} \right) \left( \frac{\partial {\alpha }_{NE}}{\partial x} \right) $ derivatives of *α*_*NE*_ with respect to parameters are as follows: *SI*_*β*_*hh*__ = 1.415, *SI*_*β*_*sh*__ = 3.472, *SI*_*β*_*ss*__ = 10.911, *SI*_*μ*_*s*__ = 0.932, *SI*_*ρ*_*s*__ =  − 7.120, *SI*_*ρ*_*h*__ =  − 1.258, *SI*_*ν*_*s*__ = 0.077, *SI*_*ν*_*h*__ = 0.004, *SI*_*d*_*s*__ =  − 5.917, *SI*_*d*_*h*__ =  − 0.141, *SI*_*C*_*V*__ =  − 3.518, *SI*_*C*_*MPX*__ = 3.603.

As shown in Fig. 2, as *α*_*h*_ increases, *C*_*notV*_(*α*_*h*_) approaches an asymptote. Consequently, the value of *α*_*NE*_, a solution to *C*_*notV*_(*α*_*h*_) = *C*_*V*_ can be very sensitive to *C*_*V*_ when *C*_*V*_ ≈ 3. Any small decrease of *C*_*V*_ can cause a significant increase of *α*_*NE*_. The same sensitivity is demonstrated in Fig. 3. Figure 4 shows the sensitivity analysis and how *α*_*NE*_ depends on variation of different parameters. We can see the high sensitivity of *α*_*NE*_ on the squirrel-to-human transmission rate, *β*_*sh*_, and on the cost of vaccination, *C*_*V*_, for low values of *C*_*V*_. Moreover, the figure demonstrates that *α*_*NE*_ can be quite sensitive on the effective transmission rate amongst squirrels, *β*_*ss*_ and the squirrels recovery rate, *ρ*_*s*_. For ${\beta }_{ss}\lt ({\mu }_{s}+{d}_{s}+{\rho }_{s})\cdot \frac{{\mu }_{s}+{\nu }_{s}}{{\nu }_{s}} $, there is a semi-endemic equilibrium and *α*_*NE*_ = 0. However, as *β*_*ss*_ increases above that threshold value, *α*_*NE*_ rapidly increases and, when *β*_*ss*_ > 45, there is no Nash equilibrium vaccination rate. Similarly, when *ρ*_*s*_ is large enough to have a semi-endemic equilibrium, the optimal vaccination rate is 0. However, for small *ρ*_*s*_, there is no Nash equilibrium and the change is relatively abrupt as in the case of *β*_*ss*_.

### Model validation

For the parameters in Table 1, the proportion of seropositive squirrels, given as $ \frac{{I}_{s}+{R}_{s}}{{N}_{s}} $, is 24.44% which generally agrees with Khodakevich, Ježek & Messinger (1988). Also, the proportion of seropositive people, $ \frac{{I}_{h}+{R}_{h}}{{N}_{h}} $, is 1.06%, again agreeing with Khodakevich, Ježek & Messinger (1988).

## Discussion

The phylogenetic relatedness between MPXV and variola virus grants the smallpox vaccine an 85% effectiveness in preventing MPX (Reynolds & Damon, 2012). Poxviruses from the *Orthopoxvirus* genus have cross-reactive antibodies, meaning that vaccinated individuals would have a much lower risk of infection and mortality compared to unvaccinated individuals (Louten, 2016). The imperfect prevention rate of the vaccine was omitted in the design of the mathematical model for the sake of simplicity. As noted in Wu, Fu & Wang (2011), imperfect protection aggravates the dilemma of voluntary protective actions as lower vaccine effectiveness can lead to better vaccine coverage and smaller free-riding effects; however, the impact of the epidemic can be harder to mitigate.

It is of interest to identify and evaluate possible preventative measures in addition to vaccination that would have a measurable effect on the transmission of MPX. For instance, decreasing the animal-to-human contact and launching an education campaign about dangers of eating raw meat which seems to be the main culprit behind squirrel-to-human transmission Khodakevich, Ježek & Messinger (1988) would significantly decrease the animal-to-human transmission rate. It could still come at a considerable individual cost (such as decrease of meat supply) but it would not require a complex or well-developed healthcare infrastructure needed for the vaccination, thus providing the general population with an easily accessible preventative measure. The mathematical model for such a measure would become more complex. The main idea would follow the spirit of Kobe et al. (2018) that investigated a situation for cholera prevention where individuals could either vaccinate or avoid drinking potentially contaminated water.

The reservoir host for monkeypox remains unclear (Di Giulio & Eckburg, 2004; Falendysz et al., 2017). We focused on a moribund rope squirrel, *Funisciurus anerythrus*, but we note that the disease has also been confirmed in other animals (Arita et al., 1985; Khodakevich, Ježek & Messinger, 1988; Reynolds et al., 2010). As noted in Falendysz et al. (2017), in a recent outbreak of MPX in DRC, no association was found between contact with rope squirrels and human infection (Nolen et al., 2015). Additionally, a recent survey of 34 villages in the Tshuapa region of DRC did not detect contact with a rope squirrel carcass in the previous 30 days, although they reported contacts with red-legged sun squirrel, *Heliosciurus rufobrachium*, (Monroe et al., 2015) which was identified in Khodakevich, Ježek & Messinger (1988) as another frequent host of monkeypox.

## Conclusions

We modeled MPX dynamics using the compartmental model of Usman & Adamu (2017). As one of our major contributions, we provided closed form formulas of the equilibrium states of the dynamics. Moreover, we also showed a potential existence of the semi-endemic equilibrium, in which there is no infection in the squirrel population and the disease still persists in the human population. Currently, MPX does not seem to have the viral fitness to become endemic solely through human transmission. Yet, simple mutations in viral proteins could still occur and increase successive inter-human cases as seen in the H1N1 virus outbreak (Le et al., 2009). Should this mutation occur, a careful understanding of the semi-endemic equilibrium will be needed.

In addition, we applied a game-theoretical approach to assess vaccination decision-making developed by Bauch & Earn (2004). Individuals in any population susceptible to MPX have the choice to vaccinate against the disease or risk the possibility of contracting the disease. Naturally, it is in the individual’s best interest to choose the option with the smaller expected cost. The model quantifies the costs and benefits of getting smallpox vaccine. We found that the optimal vaccination rate is about 0.04, i.e., individuals should vaccinate about once every 25 years.

We must note that the parameter values we used are only estimates based on available literature. In reality, the parameters may be quite different, in large part because the reservoir hosts are different as discussed above. The performed sensitivity analysis allows us to gain insight into how our results depend on the specific parameter values. We observed that the optimal vaccination rate, *α*_*NE*_, is about 10 times more sensitive to parameters related to animal hosts than to a corresponding parameter related to humans. It is therefore important to establish more accurate parameters. Consequently, greater efforts are needed to track the true prevalence and recurrent cases of MPX in all populations rather than relying on suspected cases.

Though not perfect in practice, mathematical modeling of diseases remains a powerful tool that grants a more profound understanding how MPX operates under certain conditions. The scope of epidemiological modeling and game-theoretic cost analysis is wide. As cases of MPX become increasingly reported among humans (Antwerpen et al., 2019), we hope that the models may serve as a predictive tool to better study the spread of MPX.

##  Supplemental Information

10.7717/peerj.9272/supp-1 Supplemental Information 1 Matlab code to generate figures and do the analysis

## Appendix 1. Step-by-Step Solutions to Equilibrium States

In this section we find the equilibrium states of the system (1)–(9). We will look for solutions of the following system.

(15) \begin{eqnarray*}0& ={\Lambda }_{s}- \left( {\mu }_{s}+{\beta }_{ss} \frac{{I}_{s}}{{N}_{s}} \right) {S}_{s}\end{eqnarray*}

(16) \begin{eqnarray*}0& ={\beta }_{ss} \frac{{I}_{s}}{{N}_{s}} {S}_{s}-({\mu }_{s}+{\nu }_{s}){E}_{s}\end{eqnarray*}

(17) \begin{eqnarray*}0& ={\nu }_{s}{E}_{s}-({\mu }_{s}+{d}_{s}+{\rho }_{s}){I}_{s}\end{eqnarray*}

(18) \begin{eqnarray*}0& ={\rho }_{s}{I}_{s}-{\mu }_{s}{R}_{s}\end{eqnarray*}

(19) \begin{eqnarray*}0& ={\Lambda }_{h}- \left( {\mu }_{h}+ \left( {\beta }_{sh} \frac{{I}_{s}}{{N}_{s}} +{\beta }_{hh} \frac{{I}_{h}}{{N}_{h}} \right) +{\alpha }_{h} \right) {S}_{h}\end{eqnarray*}

(20) \begin{eqnarray*}0& ={\alpha }_{h}{S}_{h}-{\mu }_{h}{V}_{h}\end{eqnarray*}

(21) \begin{eqnarray*}0& = \left( {\beta }_{sh} \frac{{I}_{s}}{{N}_{s}} +{\beta }_{hh} \frac{{I}_{h}}{{N}_{h}} \right) {S}_{h}-({\mu }_{h}+{\nu }_{h}){E}_{h}\end{eqnarray*}

(22) \begin{eqnarray*}0& ={\nu }_{h}{E}_{h}-({\mu }_{h}+{d}_{h}+{\rho }_{h}){I}_{h}\end{eqnarray*}

(23) \begin{eqnarray*}0& ={\rho }_{h}{I}_{h}-{\mu }_{h}{R}_{h}\end{eqnarray*}

where (15)–(18) are equations for the squirrels and (19)–(23) are for the humans.

We will distinguish three equilibrium states *ϵ*^0^, *ϵ*^∗^ and *ϵ*^†^ depending on the existence of infection among squirrels and humans. However, by (16), (18), (20), (21) and (23), no matter which equilibrium state, the following formulas will always be valid.

(24) \begin{eqnarray*}{E}_{s}& = \frac{{\mu }_{s}+{d}_{s}+{\rho }_{s}}{{\nu }_{s}} {I}_{s}\end{eqnarray*}

(25) \begin{eqnarray*}{R}_{s}& = \frac{{\rho }_{s}}{{\mu }_{s}} {I}_{s}\end{eqnarray*}

(26) \begin{eqnarray*}{V}_{h}& = \frac{{\alpha }_{h}}{{\mu }_{h}} {S}_{h}\end{eqnarray*}

(27) \begin{eqnarray*}{E}_{h}& = \frac{{\mu }_{h}+{d}_{h}+{\rho }_{h}}{{\nu }_{h}} {I}_{h}\end{eqnarray*}

(28) \begin{eqnarray*}{R}_{h}& = \frac{{\rho }_{h}}{{\mu }_{h}} {I}_{h}\end{eqnarray*}

### Disease-free equilibrium, ϵ^0^

Assume ${I}_{h}^{0}={I}_{s}^{0}=0$. It follows from (24) that ${E}_{s}^{0}=0$, from (25) that ${R}_{s}^{0}=0$ and from (15) that ${S}_{s}^{0}= \frac{{\Lambda }_{s}}{{\mu }_{s}} $. Also, by (27), ${E}_{h}^{0}=0$ and, by (28), ${R}_{h}^{0}=0$. It follows from (19) that

(29) \begin{eqnarray*}{S}_{h}^{0}= \frac{{\Lambda }_{h}}{{\alpha }_{h}+{\mu }_{h}} .\end{eqnarray*}

and thus, by (26),

(30) \begin{eqnarray*}{V}_{h}^{0}= \frac{{\Lambda }_{h}}{{\mu }_{h}} \cdot \frac{{\alpha }_{h}}{{\alpha }_{h}+{\mu }_{h}} .\end{eqnarray*}

The stability of *ϵ*^0^ was discussed and the basic reproduction numbers were derived in Usman & Adamu (2017) using the next-generation matrix method of Van den Driessche & Watmough (2002). Here we present an alternative derivation of the basic reproduction numbers.

Assume there is an infected squirrel in an otherwise disease-free population. The squirrel stays infected for a period of (*μ*_*s*_ + *d*_*s*_ + *ρ*_*s*_)^−1^ during which it exposes susceptible individuals at the rate ${\beta }_{ss} \frac{{S}_{s}^{0}}{{N}_{s}^{0}} ={\beta }_{ss}$. The newly exposed individuals end up in the *I*_*s*_ compartment with probability $ \frac{{\nu }_{s}}{{\mu }_{s}+{\nu }_{s}} $. Consequently, the number of secondary infections from a single infected squirrel in an otherwise disease free equilibrium is given by

(31) \begin{eqnarray*}{R}_{0ss}={\beta }_{ss}\cdot \left( \frac{1}{{\mu }_{s}+{d}_{s}+{\rho }_{s}} \right) \cdot \left( \frac{{\nu }_{s}}{{\mu }_{s}+{\nu }_{s}} \right) .\end{eqnarray*}

Similarly, we can derive that the number of secondary infections caused by a single infected human in otherwise disease-free population is

(32) \begin{eqnarray*}{R}_{0hh}& ={\beta }_{hh} \frac{{S}_{h}^{0}}{{N}_{h}^{0}} \cdot \left( \frac{1}{{\mu }_{h}+{d}_{h}+{\rho }_{h}} \right) \cdot \left( \frac{{\nu }_{h}}{{\mu }_{h}+{\nu }_{h}} \right) = \frac{{\nu }_{h}{\beta }_{hh}{\mu }_{h}}{({\mu }_{h}+{d}_{h}+{\rho }_{h})({\mu }_{h}+{\nu }_{h})({\alpha }_{h}+{\mu }_{h})} .\end{eqnarray*}

### Case when *I*_*s*_ > 0

By adding (15)–(18), we get $0={\Lambda }_{s}-{\mu }_{s}{N}_{s}^{\ast }-{d}_{s}{I}_{s}^{\ast }$ which yields

(33) \begin{eqnarray*}{I}_{s}^{\ast }= \frac{{\Lambda }_{s}-{\mu }_{s}{N}_{s}^{\ast }}{{d}_{s}} .\end{eqnarray*}

By (24), (16) becomes

(34) \begin{eqnarray*}0= \frac{{\beta }_{ss}{I}_{s}^{\ast }{S}_{s}^{\ast }}{{N}_{s}^{\ast }} -({\mu }_{s}+{\nu }_{s})\cdot \frac{{\mu }_{s}+{d}_{s}+{\rho }_{s}}{{\nu }_{s}} \cdot {I}_{s}^{\ast }\end{eqnarray*}

and since we are assuming ${I}_{s}^{\ast }\gt 0$, we can divide by *I*_*s*_ and get

(35) \begin{eqnarray*}{S}_{s}^{\ast }& ={N}_{s}^{\ast }\cdot \frac{({\mu }_{s}+{\nu }_{s})({\mu }_{s}+{d}_{s}+{\rho }_{s})}{{\beta }_{ss}{\nu }_{s}} = \frac{{N}_{s}^{\ast }}{{R}_{0ss}} .\end{eqnarray*}

Consequently,

(36) \begin{eqnarray*}{N}_{s}^{\ast }& ={S}_{s}^{\ast }+{E}_{s}^{\ast }+{I}_{s}^{\ast }+{R}_{s}^{\ast }\nonumber\\\displaystyle & = \frac{{N}_{s}^{\ast }}{{R}_{0ss}} + \left( \frac{{\mu }_{s}+{d}_{s}+{\rho }_{s}}{{\nu }_{s}} +1+ \frac{{\rho }_{s}}{{\mu }_{s}} \right) \cdot \left( \frac{{\Lambda }_{s}-{\mu }_{s}{N}_{s}^{\ast }}{{d}_{s}} \right) \end{eqnarray*}

which yields

(37) \begin{eqnarray*}{N}_{s}^{\ast }= \frac{{\Lambda }_{s}\cdot \left( \frac{{\mu }_{s}+{d}_{s}+{\rho }_{s}}{{\nu }_{s}} +1+ \frac{{\rho }_{s}}{{\mu }_{s}} \right) }{{\mu }_{s}\cdot \left( \frac{{\mu }_{s}+{d}_{s}+{\rho }_{s}}{{\nu }_{s}} \right) +{\mu }_{s}+{\rho }_{s}+{d}_{s} \left( 1- \frac{1}{{R}_{0ss}} \right) } .\end{eqnarray*}

### Fully endemic equilibrium, *ϵ**, human population

Adding (19)–(23) yields

(38) \begin{eqnarray*}0={\Lambda }_{h}-{\mu }_{h}{N}_{h}^{\ast }-{d}_{h}{I}_{h}^{\ast }\end{eqnarray*}

and consequently

(39) \begin{eqnarray*}{I}_{h}^{\ast }= \frac{{\Lambda }_{h}-{\mu }_{h}{N}_{h}^{\ast }}{{d}_{h}} .\end{eqnarray*}

Substituting (39) into (23), (21), (19), (20) we get

(40) \begin{eqnarray*}{R}_{h}^{\ast }& = \frac{{\rho }_{h}}{{\mu }_{h}} \cdot \left( \frac{{\Lambda }_{h}-{\mu }_{h}{N}_{h}^{\ast }}{{d}_{h}} \right) \end{eqnarray*}

(41) \begin{eqnarray*}{E}_{h}^{\ast }& = \left( \frac{{\mu }_{h}+{d}_{h}+{\rho }_{h}}{{\nu }_{h}} \right) \cdot \left( \frac{{\Lambda }_{h}-{\mu }_{h}{N}_{h}^{\ast }}{{d}_{h}} \right) \end{eqnarray*}

(42) \begin{eqnarray*}{S}_{h}^{\ast }& = \frac{({\mu }_{h}+{\nu }_{h})\cdot \left( \frac{{\mu }_{h}+{d}_{h}+{\rho }_{h}}{{\nu }_{h}} \right) \cdot \left( \frac{{\Lambda }_{h}-{\mu }_{h}{N}_{h}^{\ast }}{{d}_{h}} \right) }{{\beta }_{sh} \left( \frac{{I}_{s}^{\ast }}{{N}_{s}^{\ast }} \right) +{\beta }_{hh} \frac{ \frac{{\Lambda }_{h}-{\mu }_{h}{N}_{h}^{\ast }}{{d}_{h}} }{{N}_{h}^{\ast }} } \end{eqnarray*}

(43) \begin{eqnarray*}{V}_{h}^{\ast }& = \frac{{\alpha }_{h}}{{\mu }_{h}} \cdot \left( \frac{({\mu }_{h}+{\nu }_{h})\cdot \left( \frac{{\mu }_{h}+{d}_{h}+{\rho }_{h}}{{\nu }_{h}} \right) \cdot \left( \frac{{\Lambda }_{h}-{\mu }_{h}{N}_{h}^{\ast }}{{d}_{h}} \right) }{{\beta }_{sh} \left( \frac{{I}_{s}^{\ast }}{{N}_{s}^{\ast }} \right) +{\beta }_{hh} \frac{ \frac{{\Lambda }_{h}-{\mu }_{h}{N}_{h}^{\ast }}{{d}_{h}} }{{N}_{h}^{\ast }} } \right) .\end{eqnarray*}

Since ${N}_{h}^{\ast }={S}_{h}^{\ast }+{V}_{h}^{\ast }+{E}_{h}^{\ast }+{I}_{h}^{\ast }+{R}_{h}^{\ast }$, we get

(44) \begin{eqnarray*}{N}_{h}^{\ast }= & \frac{{\rho }_{h}}{{\mu }_{h}} \left( \frac{{\Lambda }_{h}-{\mu }_{h}{N}_{h}^{\ast }}{{d}_{h}} \right) + \left( \frac{{\mu }_{h}+{d}_{h}+{\rho }_{h}}{{\nu }_{h}} \right) \cdot \left( \frac{{\Lambda }_{h}-{\mu }_{h}{N}_{h}^{\ast }}{{d}_{h}} \right) & & & + \frac{{\Lambda }_{h}-{\mu }_{h}{N}_{h}^{\ast }}{{d}_{h}} + \frac{ \left( {\mu }_{h}+{\nu }_{h} \right) \cdot \left( \frac{{\mu }_{h}+{d}_{h}+{\rho }_{h}}{{\nu }_{h}} \right) \cdot \left( \frac{{\Lambda }_{h}-{\mu }_{h}{N}_{h}^{\ast }}{{d}_{h}} \right) }{{\beta }_{sh} \left( \frac{{I}_{s}^{\ast }}{{N}_{s}^{\ast }} \right) +{\beta }_{hh} \frac{ \frac{{\Lambda }_{h}-{\mu }_{h}{N}_{h}^{\ast }}{{d}_{h}} }{{N}_{h}^{\ast }} } & & + \frac{{\alpha }_{h}}{{\mu }_{h}} \cdot \left( \frac{({\mu }_{h}+{\nu }_{h})\cdot \left( \frac{{\mu }_{h}+{d}_{h}+{\rho }_{h}}{{\nu }_{h}} \right) \cdot \left( \frac{{\Lambda }_{h}-{\mu }_{h}{N}_{h}^{\ast }}{{d}_{h}} \right) }{{\beta }_{sh} \left( \frac{{I}_{s}^{\ast }}{{N}_{s}^{\ast }} \right) +{\beta }_{hh} \frac{ \frac{{\Lambda }_{h}-{\mu }_{h}{N}_{h}^{\ast }}{{d}_{h}} }{{N}_{h}^{\ast }} } \right) . \end{eqnarray*}

This yields that ${N}_{h}^{\ast }$ is a positive root of the equation $A{N}_{h}^{2}+B{N}_{h}+C=0$, i.e.,

(45) \begin{eqnarray*}{N}_{h}^{\ast }= \frac{-B+\sqrt{{B}^{2}-4AC}}{2A} \end{eqnarray*}

where

(46) \begin{eqnarray*}A& =g\cdot {d}_{h}+k\cdot c\cdot {d}_{h}\cdot {\mu }_{h} \left( 1+ \frac{{\alpha }_{h}}{{\mu }_{h}} \right) +g\cdot {\mu }_{h} \left( 1+z+k \right) \end{eqnarray*}

(47) \begin{eqnarray*}B& ={d}_{h}\cdot {\beta }_{hh}\cdot {\Lambda }_{h}- \left( g\cdot {\Lambda }_{h}-{\beta }_{hh}\cdot {\Lambda }_{h}\cdot {\mu }_{h} \right) \left( 1+z+k \right) -k\cdot c\cdot {d}_{h}\cdot {\Lambda }_{h} \left( 1+ \frac{{\alpha }_{h}}{{\mu }_{h}} \right) \end{eqnarray*}

(48) \begin{eqnarray*}C& =-{\Lambda }_{h}^{2}\cdot {\beta }_{hh} \left( 1+z+k \right) \end{eqnarray*}

and

(49) \begin{eqnarray*}k& = \frac{{\mu }_{h}+{d}_{h}+{\rho }_{h}}{{\nu }_{h}} \end{eqnarray*}

(50) \begin{eqnarray*}g& ={d}_{h}{\beta }_{sh} \left( \frac{{I}_{s}^{\ast }}{{N}_{s}^{\ast }} \right) -{\beta }_{hh}{\mu }_{h}\end{eqnarray*}

(51) \begin{eqnarray*}c& ={\mu }_{h}+{\nu }_{h}\end{eqnarray*}

(52) \begin{eqnarray*}z& = \frac{{\rho }_{h}}{{\mu }_{h}} .\end{eqnarray*}

The positive solution for ${N}_{h}^{\ast }$ from (45) can then be recursively substituted into (39), (40), (41), (42), and (43) to get closed-form formulas for equilibrium values.

### Semi-endemic equilibrium

Here, *I*_*s*_ = 0. As above, by adding all equations (19)–(23) we get $0={\Lambda }_{h}-{\mu }_{h}{N}_{h}^{\dagger }-{d}_{h}{I}_{h}^{\dagger }$ which yields

(53) \begin{eqnarray*}{I}_{h}^{\dagger }= \frac{{\Lambda }_{h}-{\mu }_{h}{N}_{h}^{\dagger }}{{d}_{h}} .\end{eqnarray*}

Substituting Eq. (53) into Eqs. (23), (21), (19) we get

(54) \begin{eqnarray*}{R}_{h}^{\dagger }& = \frac{{\rho }_{h}}{{\mu }_{h}} \cdot \left( \frac{{\Lambda }_{h}-{\mu }_{h}{N}_{h}^{\dagger }}{{d}_{h}} \right) \end{eqnarray*}

(55) \begin{eqnarray*}{E}_{h}^{\dagger }& = \left( \frac{{\mu }_{h}+{d}_{h}+{\rho }_{h}}{{\nu }_{h}} \right) \cdot \left( \frac{{\Lambda }_{h}-{\mu }_{h}{N}_{h}^{\dagger }}{{d}_{h}} \right) \end{eqnarray*}

(56) \begin{eqnarray*}{S}_{h}^{\dagger }& = \frac{({\mu }_{h}+{\nu }_{h})\cdot \left( \frac{{\mu }_{h}+{d}_{h}+{\rho }_{h}}{{\nu }_{h}} \right) \cdot \left( \frac{{\Lambda }_{h}-{\mu }_{h}{N}_{h}^{\dagger }}{{d}_{h}} \right) }{{\beta }_{sh} \left( \frac{{I}_{s}^{\dagger }}{{N}_{s}^{\dagger }} \right) +{\beta }_{hh} \frac{ \frac{{\Lambda }_{h}-{\mu }_{h}{N}_{h}^{\dagger }}{{d}_{h}} }{{N}_{h}^{\dagger }} } .\end{eqnarray*}

Since ${I}_{s}^{\dagger }=0$, we get

(57) \begin{eqnarray*}{S}_{h}^{\dagger }={N}_{h}^{\dagger }\cdot \frac{ \left( {\mu }_{h}+{d}_{h}+{\rho }_{h} \right) \left( {\mu }_{h}+{\nu }_{h} \right) }{{\nu }_{h}{\beta }_{hh}} \end{eqnarray*}

and consequently, by (20),

(58) \begin{eqnarray*}{V}_{h}^{\dagger }={N}_{h}^{\dagger }\cdot \left( \frac{{\alpha }_{h}}{{\mu }_{h}} \right) \cdot \frac{ \left( {\mu }_{h}+{d}_{h}+{\rho }_{h} \right) \left( {\mu }_{h}+{\nu }_{h} \right) }{{\nu }_{h}{\beta }_{hh}} .\end{eqnarray*}

Finally, summing up (53)–(58), we obtain the closed-form formula

(59) \begin{eqnarray*}{N}_{h}^{\dagger }= \frac{{\Lambda }_{h}\cdot \left( \frac{{\rho }_{h}}{{\mu }_{h}} + \frac{{\mu }_{h}+{d}_{h}+{\rho }_{h}}{{\nu }_{h}} +1 \right) }{{d}_{h}+{\mu }_{h}\cdot \left( \frac{{\rho }_{h}}{{\mu }_{h}} + \frac{{\mu }_{h}+{d}_{h}+{\rho }_{h}}{{\nu }_{h}} +1 \right) -{d}_{h}\cdot \left( \frac{{\mu }_{h}+{d}_{h}+{\rho }_{h}}{{\nu }_{h}} \left( \frac{{\mu }_{h}+{\nu }_{h}}{{\beta }_{hh}} \right) + \frac{{\alpha }_{h}}{{\mu }_{h}} \left( \frac{{\mu }_{h}+{d}_{h}+{\rho }_{h}}{{\nu }_{h}} \right) \left( \frac{{\mu }_{h}+{\nu }_{h}}{{\beta }_{hh}} \right) \right) } \end{eqnarray*}

which can be used to calculate equilibrium values.

## Additional Information and Declarations

### Data Availability

The following information was supplied regarding data availability: The Matlab code used to generate the figures is available as a Supplemental File.
